# Current Issues in Atrial Fibrillation

**DOI:** 10.5402/2012/376071

**Published:** 2012-06-21

**Authors:** Yaariv Khaykin, Yana Shamiss

**Affiliations:** ^1^Heart Rhythm Program, Southlake Regional Health Centre, 105-712 Davis Drive, Newmarket, ON, Canada L3Y 8C3; ^2^Faculty of Medicine, University of Toronto, Toronto, ON, Canada M5S 1A8

## Abstract

Atrial fibrillation (AF) is the most common sustained cardiac arrhythmia. It places an enormous burden on the patients, caregivers, and the society at large. While the main themes in the care of an AF patient have not changed over the years and continue to focus on stroke prevention, control of the ventricular, rate and rhythm maintenance, there have been a number of new developments in each of these realms. This paper will discuss the “hot” topics in AF in 2012 including new and upcoming medical and invasive management strategies for this condition.

## 1. Introduction

*“There is no other serious cardiac disorder which can be so speedily benefited as the well-managed case of auricular fibrillation…the most reliable preparation to use is a fresh and known tincture of digitalis.” *

This quote from *Clinical Disorders of the Heart Beat* by Lewis published in 1925 [1] could not be further from the truth in 2012. With the multitude of new therapies introduced and in development to address various clinical implications of this most common sustained rhythm disorder, it is becoming a daunting task to select the right approach to each individual patient. 

Atrial fibrillation (AF) is responsible for most arrhythmia-related hospital admissions [2] and is the most common cause of ischemic stroke [3]. Furthermore, AF carries a tremendous negative impact on the quality of life and is associated with increased mortality [4]. Its prevalence is rising in our ageing society [5, 6] and so does the expense related to its management [7] and productivity lost among the suffering patients [8].

Decisions that need to be made in each AF patient care include selection of rhythm control or the more conservative control of the ventricular rate and selection of stroke prevention strategy. At each step the clinician needs to decide between medical and invasive solutions. Since AF is frequently associated with other comorbid conditions, these need to be addressed as well.

Clinical practice guidelines developed by various professional societies attempt to help physicians select the right therapies for the right AF patients. Unfortunately, the many nuances of AF presentation and available therapies complicate clinical decision-making, guidelines lag behind new clinical developments, and few mechanisms are in place to translate guidelines into standards of care. The purpose of this paper is to discuss the “hot” topics in AF care in 2012.

## 2. Preventing Embolic Sequelae

Prevention of embolic complications is the most important aspect of care for AF patients. These range from transient ischemic events (TIAs) to strokes and are the most costly complication of atrial fibrillation. Strokes secondary to AF are more severe than those secondary to atherosclerotic disease and impart a greater disability on the victims [7]. This results in significant costs related to hospitalizations, rehabilitation, and chronic disability. Strategies aimed at reducing embolic events in AF patients include therapy with aspirin, combination of aspirin and clopidogrel, and oral anticoagulation therapy with warfarin or one of the new agents targeting either thrombin or Factor Xa [9–11]. 

Despite all of this, currently as few as 10–20% of the AF patients are treated with appropriate prophylaxis strategies [12]. Those who do take warfarin spend much of their time taking subtherapeutic doses of the medication placing them at risk of stroke, while others take supertherapeutic doses and run a significant risk of bleeding, given a very narrow therapeutic range of this drug. Novel antithrombotic agents allow for more consistent anticoagulation and have been shown superior to warfarin in stroke prevention. 

In the open-label study of Dabigatran versus Warfarin in Patients with Atrial Fibrillation (RELY) the use of dabigatran, a direct thrombin inhibitor, was associated with similar rates of stroke and systemic embolism but lower rate of major bleeding compared to warfarin at a lower dose of 110 mg, while the higher dose of the drug at 150 mg was associated with 35% lower rates of stroke and systemic embolism but similar rates of major bleeding compared to warfarin [11]. Similarly, in the blinded study of Apixaban versus Warfarin in Patients with Atrial Fibrillation (ARISTOTLE), apixaban, a factor Xa inhibitor, was superior to warfarin in preventing stroke or systemic embolism with a 21% relative risk reduction in this outcome. Apixaban caused less bleeding with a relative risk reduction of over 30% and lowered all-cause mortality by 11% [13]. 

 Rivaroxaban, another factor Xa inhibitor, was studied in a somewhat different setting than the other two agents. While most patients in the RELY and ARISTOTLE studies were at a relatively low risk for stroke, in the rivaroxaban once daily oral direct factor Xa inhibition compared with vitamin K antagonism for prevention of stroke and Embolism Trial in Atrial Fibrillation (ROCKET-AF) study, only 10% of the patients had CHADS-2 score less than 3 with the remainder of the patients at higher risk of stroke. Unlike the other two agents, rivaroxaban was shown to be noninferior to warfarin in these patients in the intention-to-treat analysis but reduced stroke or systemic embolism by 21% among patients actually taking the drug [14]. All of these agents are renally eliminated to some extent requiring dose adjustment among patients with impaired renal clearance particularly for dabigatran and rivaroxaban. INR was in therapeutic range only 64% of the time in RELY, 62% of the time in ARISTOTLE, and 55% of the time in ROCKET-AF. While subanalyses of the RELY and ROCKET-AF studies showed benefit of the new agents compared to patients spending more time with INR in therapeutic range, these were post hoc analyses, raising some uncertainty about the benefit of these agents in patients who are already well controlled on warfarin [15, 16]. 

While effective from the point of view of preventing strokes and other embolic events, one must be aware of significant risk of bleeding associated with these agents used alone and, especially, in combination. Unfortunately neither has a specific reversal agent. It appears possible to remove dabigatran using hemodialysis and to reverse the other two agents with prothrombin complex concentrate which has no effect on the anticoagulant effect of dabigatran [17]. All agents showed a significant decrease in the dreaded intracranial hemorrhage compared to warfarin, but there was a trend to increased gastrointestinal bleeding with rivaroxaban and with the higher dose of dabigatran at 150 mg twice per day.

One other strategy aiming to minimize the risk of embolic events involves mechanical elimination or closure of the left atrial appendage (LAA), the area where clots related to atrial fibrillation most commonly form. Techniques for LAA closure or excision have been initially developed by the cardiac surgeons [18]. Novel LAA closure devices have recently shown promise in reducing the risk of stroke in patients who cannot take antithrombotic agents and can be placed percutaneously [19]. In a large randomized study, these devices were shown to be non-inferior to warfarin with slight excess of periprocedural complications in the invasive arm of the study [20].

With “crowding” in the area of embolic prevention, selection of the best approach for the patient is becoming more problematic [19]. Several scores have been developed to predict both the risk of stroke and that of significant bleeding [21–23] yet the guidelines incorporating those scores provide little guidance among the low-to-intermediate risk patients with guideline “creep” towards recommending OAC for a greater stratum of patients, despite little new data on the risk of stroke, lower stroke risk for the same risk score in some recently reported studies [24], and evidence that combination of aspirin and clopidogrel is equivalent in stroke prevention to warfarin, provided patients spend 65% of the time or less in therapeutic range [25], a figure higher than that for the warfarin-treated arm of all studies showing equivalent or greater efficacy of the novel antithrombotic agents. Nevertheless, several professional organizations charged with creating practice guidelines rapidly adopted the novel agents as a standard of care, in some cases before the agents were actually approved for market use [26, 27].

Guidance to avoid anticoagulation in patients at high risk of bleeding makes therapeutic selection even more cumbersome with many of the risk factors listed as associated with increased bleeding rate also present in the risk scores used to estimate the hazard of thromboembolic events [23, 28].

Nevertheless, availability of the new oral anticoagulation agents with faster onset of action and better-defined kinetics as well as left atrial appendageal occlude devices will help standardize care of AF patients and make it less arduous. These innovations will help clinicians get away from the perils of bridging anticoagulation around invasive procedures in high-risk patients and prevent or shorten hospital admissions. Time and further studies will help better define the best of the new agents and devices and, perhaps, tailor therapy to individual patient specifics.

## 3. Rate, Rhythm, or Quality of Life?

From the outset of clinical investigation into AF management it made common sense to pursue normal sinus rhythm as the goal for most patients. It seemed only natural that patients in sinus rhythm should fare better than those in AF. A number of studies set out to compare outcomes in patients treated with the goal to achieve sinus rhythm or remain in atrial fibrillation with a controlled ventricular response. As a surprise to many, these studies uniformly showed little advantage to the strategy of rhythm maintenance. 

In Pharmacological Intervention in AF (PIAF) study, 252 patients with persistent AF were randomly assigned to diltiazem for rate control or amiodarone for maintenance of sinus rhythm [29]. All patients were anticoagulated to prevent embolic sequelae of the arrhythmia. There was no difference in the Quality of Life (QOL) measures at 1 year with some functional capacity improvement in patients treated with amiodarone at a cost of more hospital admissions in this group. 

Strategies of Treatment of AF (STAF) randomized 200 patients with chronic AF, large left atria, heart failure symptoms, and mild-to-moderate LV dysfunction to amiodarone or a Class I antiarrhythmic drug versus rate control [30]. All patients were anticoagulated. While there was no difference in the likelihood of the composite endpoint of death or embolic event, there was a trend to higher risk of stroke in the rhythm control arm. 

Rate Control versus Electrical Cardioversion (RACE) study randomized 522 patients with persistent AF to antiarrhythmic therapy with Sotalol, Class I medications or amiodarone versus rate control [31]. Patients in the rhythm control arm were anticoagulated only during the pericardioversion period. Clinical outcomes between the two strategies were similar at the conclusion of the trial. 

The largest of the rate versus rhythm control studies, Atrial Fibrillation Follow-up Investigation of Rhythm Management (AFFIRM) sought to compare these strategies in 4060 patients with recurrent AF [32]. Anticoagulation could be stopped in the rhythm control arm if sinus rhythm were present for at least 4 weeks. Intention-to-treat analysis of the AFFIRM study demonstrated a trend to a lower combined endpoint of mortality, embolism, and hospitalization in the rate control arm of the trial. 

Patients with congestive heart failure (CHF) would have been expected to derive the greatest benefit from sinus rhythm. These patients may be particularly sensitive to loss of atrial contraction and rapid ventricular rates, both interfering with left ventricular filling, already impaired in CHF. Nevertheless, in the Rhythm Control versus Rate Control for Atrial Fibrillation and Heart Failure (AF-CHF) study patients treated with amiodarone to achieve sinus rhythm fared no better than those treated with beta blockers and digoxin to achieve adequate rate control [33].

Findings of these studies are easier to interpret in the light of the limited efficacy of the rate and particularly rhythm control regimens. Only 38% of the patients in the rhythm control arm in STAF and 39% of the patients assigned to rhythm control in RACE were in sinus rhythm. Patients in PIAF and AFFIRM had a slightly better response to therapy, but still, only two-thirds of the patients treated with amiodarone in PIAF were in sinus rhythm, on par with 63% percent of the patients assigned to rhythm control in AFFIRM and 73% of these patients in AF-CHF who were in sinus rhythm at the conclusion of the study. By comparison, two thirds of the rate control patients in PIAF and 80% of the rate control patients in AFFIRM and AF-CHF achieved prespecified ventricular response rates. 

While the intention-to-treat analysis in RACE showed no difference in the QOL outcomes between patients assigned to rate or rhythm control, patients in sinus rhythm had better QOL in this study[34]. This was supported by the Canadian Trial of Atrial Fibrillation [35] where summary measures of physical and mental health improved significantly with treatment from baseline, regardless of therapy selection, with global well-being rated significantly lower by patients with recurrent AF compared to those without. Rhythm control strategy leads to better exercise tolerance and functional capacity in several trials, including the AFFIRM in-rhythm analysis [36]. Furthermore, when STAF investigators compared baseline quality of life measures in their subjects with those in background German population in sinus rhythm they found all SF-36 measures to be significantly lower among the study subjects. 

Patients who were actually able to achieve sinus rhythm in AFFIRM also had a 47% mortality risk reduction compared to those who were in AF (*P* < 0.0001) [37]. These findings were supported by meta-analyses of rate versus rhythm control trials [38, 39].

Why then did the patients who were treated with antiarrhythmic agents perform so poorly even though sinus rhythm itself was associated with superior outcomes? Part of the answer comes from the limited efficacy of these agents to actually control rhythm as described. The second part has to do with a significant adverse event profile of these medications and in particular amiodarone. All of these medications have been shown to increase mortality in certain populations, in particular among patients with ischemic heart disease and myocardial dysfunction, in the case of Sotalol [40] and Class IC agents [41], and among patients with left ventricular dysfunction and history of congestive heart failure, in the case of dronedarone [42, 43], a newcomer to this class of drugs. Amiodarone, while the most effective antiarrhythmic of all, has been shown to cause significant bradycardia in 5% of the patients, thyroid toxicity in 23%, skin toxicity in up to 75%, neurologic toxicity in up to 30%, and corneal deposits in 100% of the treated patients [44]. Uniformly, close to 30% of the patients treated with amiodarone in these studies stopped the drug due to side effects. 

Dronedarone, a Class III antiarrhythmic agent, was engineered based on the amiodarone molecule in hope of providing equivalent clinical benefit without multiple side effects [45]. Pharmacologically, the molecule of dronedarone does not carry iodine, thought to account for most of the end-organ toxicity seen among amiodarone patients. It was also modified to make it more hydrophilic and to expedite elimination half-life compared to its parent drug. Dronedarone had undergone extensive clinical testing in multiple trials and was shown to have rhythm control efficacy comparable to that of Sotalol or Class I C agents. In addition dronedarone has been shown to provide a measure of rate control, lowering heart rate in atrial fibrillation among treated patients by an average of 14 beats per minute [46]. Unlike other antiarrhythmic agents, dronedarone could be started on an outpatient basis without the need for in-patient monitoring required for Sotalol, Propafenone, Flecainide, and Dofetilide. ATHENA, a double-blind placebo-controlled trial, studied the effects of dronedarone in addition to standard therapy in patients with risk factors including age over 75, or age under 75 with at least one of hypertension, diabetes, stroke or TIA, enlarged left atrial dimension (>50 mm), or reduced left ventricular ejection fraction (<40%) [47]. Dronedarone was shown to reduce AF-related hospitalizations with a hazard ratio of 0.626 compared to placebo [48]. It also significantly reduced duration of hospitalization in these patients. These effects would potentially reduce the cost of care by 2875 Euro per patient per year based on the European health economics data or approximately $3000–6000 based on similar US and Canadian data. 

Dronedarone was quickly elevated to the status of first-line agent by some professional organizations [49], but soon proved neither nearly as effective as amiodarone [50, 51], nor as safe as it was touted to be [52, 53]. In a recently published Permanent Atrial Fibrillation Outcome Study Using Dronedarone on Top of Standard Therapy (PALLAS), when administered to patients with high-risk permanent atrial fibrillation dronedarone was associated with doubling of mortality among treated patients, more than double the risk of stroke and nearly double the risk of heart failure hospitalization [43]. Unfortunately, guideline production cycle has not caught up to the recent developments, leaving clinicians in a quandary. 

Several new agents are undergoing studies to determine their place in the antiarrhythmic line-up. Two of these, vernakalant and ranolazine, have substantial data behind them. Vernakalant predominantly blocks atrial potassium currents. Intravenous form of this medication has been shown to be effective in restoring sinus rhythm without significant proarrhythmic effect [54–56]. Ranolazine is a multichannel blocker approved for treatment of chronic angina. It has been shown to reduce the burden of atrial fibrillation and to have a synergistic effect with amiodarone and dronedarone [57–59].

Overall, medical therapy, whether aimed at control of the ventricular rate or rhythm, fails miserably and is poorly tolerated, in other words, medical rhythm control and rate control strategies are equally poor, rather than equally effective. Patients in AFFIRM and other medical rate versus rhythm control studies were not necessarily symptomatic—these latter patients were more likely excluded and offered rhythm control therapy. This enrolment bias further limits applicability of the findings of these studies to the clinical care of patients frequenting emergency rooms because of disabling symptoms stemming from their AF. Accordingly, selection of therapy in these patients should really focus on symptom improvement and quality of life rather than rate or rhythm control per se. 

## 4. Is the Strategy of Ablate and Pace a Good Answer to Medical Therapy for All Patients?

Advent of ablation and cardiac rhythm devices signaled renewed hope for the victims of AF and added to the armamentarium of the treating clinician. These therapies could be applied for both rate and rhythm control indications. In the first instance, AV nodal ablation and permanent pacing significantly reduced cardiac symptoms, while improving exercise duration, quality of life, and ejection fraction, in a meta-analysis of clinical studies based on over 1000 patients [60]. In particular patients with chronic AF and congestive heart failure symptoms treated with AV nodal ablation derive a greater benefit from biventricular pacing with statistically significant improvement in functional capacity, when compared with RV pacing, as measured by the 6-minute walking test, peak VO2, and exercise duration [61]. 

Some of the explanation for the greater benefit of AV junction ablation approach come from the Rate Control Efficacy in Permanent AF (RACE II) trial of aggressive versus lenient rate control strategies. Aggressive strategy in this study targeted conventional rate control criteria used in AFFIRM of resting heart rate less than 80 bpm and peak heart rate with moderate exercise of less than 110 bpm, while the lenient strategy targeted resting heart rate less than 110 bpm. In this study, patients assigned to strict heart rate control did substantially worse with a 16% greater risk of the composite of cardiovascular death, heart failure hospitalization, stroke, systemic embolism, bleeding, or “life-threatening arrhythmic events” defined as syncope, sustained VT, cardiac arrest, life-threatening adverse events of rate-control drugs, or pacemaker or ICD implantation, driven by greater rates of bradycardia-related complications. Moreover 45% of the patients in both groups still had significant AF-related symptoms at the end of the study [62].

Ablate and pace approach is very effective among the older patients with tachycardia-bradycardia syndrome who are intolerant or have contraindications to a number of medications unfortunately, this strategy is frequently applied to younger patients with greater burden of device-related complications over the lifetime. Patients thus treated may require device replacement and upgrade down the road, some may develop RV-pacing-mediated cardiomyopathy, yet others might suffer a worse fate, succumbing to sudden cardiac death as a result of pacing system failure or overly rapid down titration of the ventricular rate and ensuing ventricular arrhythmia [63]. Unlike selection of medical therapy for patients with symptomatic AF, quality of life notwithstanding, downstream considerations should guide selection of this approach.

## 5. To Burn or Not to Burn?

Catheter ablation has rapidly moved to the mainstream of AF therapy over the past decade. This approach is based on the notion that paroxysmal AF episodes arise as a result of focal firing in the pulmonary veins and elsewhere in the left and right atria [64]. While initially considered “curative,” over the last few years it is becoming apparent that many patients treated in this fashion return with further episodes of arrhythmia down the road [65], and many more continue to experience asymptomatic episodes of arrhythmia [66]. Nevertheless, studies of AF ablation have uniformly found improved quality of life among ablated patients. 

A retrospective study in 1171 consecutive patients in their mid-sixties referred with symptomatic AF was one of the first to address QOL improvement with ablation compared to medical therapy [67]. Circumferential pulmonary vein ablation was performed in 589 patients with the remainder treated medically, aiming at rhythm control. At 900 days of followup, patients treated with ablation had a 70% reduction in the likelihood of AF recurrence. Apart from an impressive improvement in survival among the ablated patients, the investigators reported similar changes in the QOL measures in both groups, with AF recurrences associated with significant reductions in physical and mental functioning in the medical group and impaired psychological well-being among the ablated patients. Similar improvements in survival and reduction in the risk of stroke among ablated patients were seen in a registry of patients treated in UK and Australia when compared to those treated medically in the Euro-Heart Survey [68]. 

A randomized trial of Radiofrequency Ablation versus Antiarrhythmic Drugs as First-line Treatment of Symptomatic Atrial Fibrillation (RAAFT) enrolled 70 patients in their mid-fifties with paroxysmal AF in 2001-2002 [69]. Within one year of followup, AF recurrence was five times less likely in patients randomized to ablation compared to those treated medically. Improvement in the QOL measures was more pronounced in the invasive arm of the study in five out of eight subclasses of the SF-36 scale.

While most patients undergoing AF ablation are young with little structural heart disease and largely paroxysmal AF, this therapy has been applied to patients with structural heart disease as well as those with persistent and chronic AF. In a study by Chen and colleagues, 94 patients with ejection fraction below 40% were ablated at the Cleveland Clinic between 2000 and 2003 [70]. After the first procedure, 73% of the patients with low ejection fraction were free of AF recurrence at 14 ± 6 months. Six months after the procedure, significant improvement in the areas of general health, energy, physical functioning, and emotional well-being was reported by the patients.

Hsu and colleagues studied 58 consecutive patients with ejection fraction less than 45% and clinical congestive heart failure symptoms [71]. After 12 ± 7 months, 78% of these patients remained in sinus rhythm with 21 ± 13% improvement in ejection fraction, even though rate control prior to ablation was considered adequate in these patients. Restoration and maintenance of sinus rhythm along with cardiac structural improvement in these patients were paralleled by improvement in measures of QOL and functional performance with NYHA functional class improving on average by one grade 12 months following the procedure. Improvement was seen in the areas of physical and mental function, as well as exercise time and capacity.

Looking at this data it may appear that just about anyone may benefit from this approach. The truth is that AF ablation primarily targets pulmonary vein antra—a known culprit responsible for arrhythmia among patients with paroxysmal AF. It should not come as a surprise that patients with persistent and chronic AF while frequently ablated do not do as well as their paroxysmal counterparts, with about double the rate of long-term failure of the procedure [65]. 

A number of studies looked at additional ablation strategies divided into those that create linear lesions between the veins and extending from the veins to the mitral annulus. In a study by Earley and colleagues, 42 patients with permanent AF were treated with circumferential pulmonary vein ablation with isolation verified using noncontact mapping approach [72]. In addition to isolating the veins, the investigators delivered linear lesions across the left atrial roof and the cavotricuspid isthmus. These procedures were time consuming at close to 5 hours and required extensive fluoroscopy despite 3D mapping. 52 % of the patients had to have a redo ablation with 74% success after multiple procedures. In a larger randomized study 142 patients received amiodarone and had a cardioversion with or without antral ablation [73]. The authors reported 74% success of the ablation approach at 12 months with 32% of the patients requiring repeat ablation. Only 3% of the patients assigned to amiodarone and a cardioversion alone maintaining sinus rhythm.

Another approach involves ablation of the areas demonstrating continuous fractionated electrical (CFE) activity during atrial fibrillation. While successful in the hands of some operators with 66% freedom from AF at one year off antiarrhythmic drugs (30% of the patients requiring a second ablation to achieve this goal) [74], this approach is difficult to reproduce because of great interoperator variability in defining target electrograms as well lack of a consistent ablation endpoint across studies. So in a study of 66 patients with LA CFE ablation only 22% converted to sinus rhythm with this approach [75]. These patients experienced 70% long-term freedom from AF. On the contrary, patients remaining in AF after CFE ablation in the left atrium who were then randomly assigned to cardioversion or additional CFE ablation in the coronary sinus and the right atrium experienced a low success rate under 30% in both groups. More recent work employing automated standardized software algorithms to identify the substrate have borne results reproducible across multiple centers and supported by at least two meta-analyses [76–78].

The other line or work aimed to identify neural mechanisms responsible for triggering and sustaining AF particularly in patients with a more persistent arrhythmia. Here, parasympathetic ganglia adjacent to the left atrium were shown in bench research to mediate AF [79]. While in an elegant animal study ablation of these ganglia was sufficient in terminating AF and rendering it noninducible [80], human data is limited primarily to an approach combining pulmonary vein antrum isolation with ablation targeting parasympathetic ganglionic plexi [81].

Some operators have included the right atrium and the coronary sinus in the lesion set. In one such study, ablation in the right atrium combined a posterior intercaval line with a septal line and SVC isolation. The results were startling, with over 80% success with the combined approach compared to 60% success with left atrial lesions alone [82].

Obviously, grouping of strategies into a prescribed “one size-fits-all” approach is too simplistic when applied to patients with chronic atrial fibrillation, likely stemming from a variety of mechanisms, not uniformly distributed across treated patients. A more intuitive approach, which has been gaining popularity, involves stepwise ablation of several targets with endpoint of AF conversion and arrhythmia termination [83]. First, pulmonary vein antra are isolated, then extensive defragmentation takes place on the roof, septum, and floor of the left atrium as well as at the base of the left atrial appendage. Finally, ablation of the mitral isthmus takes place with documentation of bidirectional block across all of the linear ablation sets. If the arrhythmia converts from AF into an atrial tachycardia or flutter, this is mapped and ablated. Ablation may also involve targets throughout the coronary sinus and in the right atrium, such as the SVC, the cavotricuspid isthmus, interatrial septum, and the crista terminalis. Using this approach AF has been shown to convert to either sinus rhythm or a more organized tachyarrhythmia in over 87% of the patients with 95% 1-year freedom from arrhythmia after 1.4 procedures/patient.

AF ablation is not without risk. While reported in each individual study, the likelihood of experiencing a complication has been best illustrated in the Worldwide Survey of Atrial Fibrillation Ablation [84]. The authors demonstrated total risk of serious complication to total 4.5% with 25 (0.15%) deaths, 213 (1.31%) tamponade, and 152 (0.94%) strokes or TIAs in 20825 catheter ablation procedures performed in 16309 patients across 182 centers. Subject to recall bias, these findings come very close to those of a recently published study of AF ablation outcomes based on an insurance database from California, where 5% of the patients had periprocedural complications [85]. The authors reported 1 death (0.02%), 13 strokes or TIAs (0.31%), and 104 (2.5%) tamponades among 4156 patients undergoing ablation “in the real world”. Unlike the negative publicity towards AF ablation that was evoked in the media by this paper, the findings are indeed positive, suggesting that AF ablation is as safe as we believed it to be despite relatively little individual center experience at a mean of 15.4 procedures per center per year in the study.

Most of the studies to date have reported outcomes of solid tip catheter ablation or ablation using a saline-irrigated tip catheter. A number of new technologies are rapidly becoming available to simplify AF ablation by allowing for energy delivery in a circumferential fashion around PV ostia. These include the PVAC catheter (Ablation Frontiers, Medtronic Inc., Minneapolis, Minnesota), utilizing duty-cycled multipolar RF energy delivery, Mesh Ablator (Bard) using unipolar RF and cryoballoon ablation (Arctic Front, Medtronic Inc., Minneapolis, Minnesota). The principal driver for these developments is the hope that the new technologies will decrease procedure time and the need for fluoroscopy, maintain or improve procedural efficacy and safety, and make AF ablation more accessible with shorter learning curves. 

Two recently published single-centre randomized studies compared AF ablation using PVAC with wide-area circumferential pulmonary vein ablation guided by Ensite Navx in one study and the CARTO system in the other [86, 87]. Both studies showed equivalent clinical outcomes with shorter procedure, radiofrequency, and fluoroscopy times achieved in patients treated with multipolar ablation. In a nonrandomised comparison of the two approaches currently in press, Tivig et al. [88] ablated 420 patients, 209 of them using PVAC. Duty-cycled RF was delivered on the septum and elsewhere in the left atrium using other multipolar ablation catheters in this group. Similarly, more extensive ablation was performed on patients treated historically at the investigators'center using 3D guided approach. This involved ablation of the roof and the mitral isthmus lines without seeking proof of block across these lines in all patients and CFE ablation among patients with persistent AF. The authors found both techniques equivalent with respect to long-term outcomes and procedural characteristics. 

One must be cautious interpreting these results and consider recent evidence suggesting that ablation using duty-cycled multipolar RF may lead to a greater cerebral ischemic burden compared to conventional irrigated ablation. In a study reported by Herrera Siklódy et al. [89], 37.5% of the patients ablated using PVAC had new cerebroembolic lesions detected by acute postprocedural magnetic resonance imaging, compared to 7.4% of the patients ablated using irrigated point-by-point RF energy delivery and 4.3% of the patients undergoing balloon cryoablation; many of these lesions could still be seen months following ablation [90]. This was supported by the findings reported by Gaita et al. (38.9% with PVAC versus 8.3% with irrigated RF and 5.6% with balloon cryoablation [91]). These concerns were recently rekindled in the context of the Tailored Treatment of Permanent Atrial Fibrillation (TTOP-AF) study presented at the 12th International Workshop on Cardiac Arrhythmias in Venice in October 2011, where 4 (2.9%) of the 138 patients ablated using this technology had full-blown clinical strokes, a much greater risk than that reported in other studies.

All in all, although not perfect, ablation of AF appears to be effective in terms of improving quality of life, particularly among patients with paroxysmal atrial fibrillation, with an acceptable safety profile. New ablation protocols and technologies will hopefully lead to improved outcomes over time, but should be adopted cautiously and after a thorough evaluation in rigorous clinical trials. More intense monitoring following the procedure may help us understand the true burden of recurrent AF following ablation and routine application of validated QOL scales to evaluate patients at every step of the way will better describe the degree of symptomatic improvement experienced by the patients. Studies into whether or not ablation results in improved morbidity or mortality among treated patients are currently on the way. 

## 6. Conclusions

Atrial fibrillation is a common condition with numerous clinical implications. Medical and invasive strategies in AF care are evolving rapidly and may be difficult to follow for the front-line clinician. Further studies are on the way to address controversies surrounding some of these approaches. In this environment, while it is very important for the professional organizations to rapidly review and adjust clinical practice guidelines as new therapies become available. Mechanisms to rapidly distribute these guidelines to the clinicians must be firmly in place for the innovation to translate into better standards of care along with safeguards precluding premature adoption of therapies. 

## Abbreviations

AF: Atrial fibrillation
LA: Left atrium
PV: Pulmonary vein
PVAI: Pulmonary vein antrum isolation
QOL: Quality of life
OAC: Oral anticoagulation
TIA: Transient ischemic attack
CHF: Congestive heart failure
CFE: Continuous (complex) fractionated electrograms
INR: International normalized ratio.
